# Dynamic Perioperative Risk Stratification of In-Hospital Mortality After Elective Isolated On-Pump CABG: A Single-Center Firth Penalized Logistic Regression Analysis

**DOI:** 10.3390/jcm15187202

**Published:** 2026-09-16

**Authors:** Anca Drăgan, Adrian Ştefan Drăgan

**Affiliations:** 1 Department of Cardiovascular Anaesthesiology and Intensive Care, “Prof. Dr. C.C. Iliescu” Emergency Institute for Cardiovascular Diseases, 258 Fundeni Road, 022328 Bucharest, Romania; 2 Faculty of General Medicine, Carol Davila University of Medicine and Pharmacy, 8 Eroii Sanitari Blvd, 050474 Bucharest, Romania; adrian-stefan.dragan2023@stud.umfcd.ro

**Keywords:** dynamic perioperative risk assessment, isolated CABG, VIS, EuroSCORE II, NLR

## Abstract

**Background**: Accurately assessing perioperative risk after elective on-pump coronary artery bypass grafting (CABG) is challenging, especially when it comes to identifying patients at high risk for early mortality. We explored whether including perioperative biochemical and hemodynamic variables could improve established preoperative risk evaluation. **Methods**: We analyzed 451 consecutive patients who underwent elective on-pump isolated CABG over two years, focusing on in-hospital mortality as the primary endpoint. The predictors were selected based on univariable analysis, clinical relevance, and multicollinearity assessment. To address the low number of mortality events, we used Firth-penalized logistic regression. The final model was assessed using discrimination, calibration, and internal validity using receiver operating characteristic analysis, calibration measures, and 1000-bootstrap resampling. A dataset-derived risk threshold was explored for risk stratification. **Results**: Eleven patients (2.44%) died during hospitalization. The final multivariable model included three variables independently associated with in-hospital mortalityEuroSCORE II (OR1.57, CI 95%: 1.13–2.14, *p* = 0.011), early postoperative vasoactive-inotropic score (VIS) (OR1.017, CI 95%: 1.008–1.033, *p* < 0.001) and preoperative CK-MB/CK ratio (OR21.06, CI 95%: 1.06–355, *p* = 0.046). The final model demonstrated good discrimination with an apparent AUC of 0.908 and an optimism-corrected AUC of 0.892. A dataset-derived risk-stratification threshold of predicted mortality ≥2.35% identified the highest-risk quintile, which contained 10 of the 11 observed deaths. Observed mortality was 11.1% in this quintile versus 0.28% among the remaining patients. Inflammatory indices showed limited predictive performance. Additionally, the use of an intra-aortic balloon pump was not independently associated with in-hospital mortality after adjusting for baseline risks. **Conclusions**: A dynamic model integrating preoperative risk assessment (EuroSCORE II and preoperative CK-MB/CK ratio) with early postoperative hemodynamic data (VIS) showed encouraging internal discrimination and calibration for mortality prediction after isolated on-pump CABG. Given the limited number of events and substantial uncertainty surrounding the CK-MB/CK ratio estimate, these findings should be considered exploratory and hypothesis-generating until externally validated.

## 1. Introduction

Coronary artery disease (CAD) is the most widespread heart condition and the leading cause of death globally, with the highest prevalence reported in Central Europe, Eastern Europe, and Central Asia [1]. This disease poses a considerable challenge to global health and places significant strain on national health systems. Coronary artery bypass grafting (CABG) remains a major approach of coronary revascularization, particularly for patients with multivessel disease and complex anatomical conditions [2]. Recent remarkable advancements, driven by innovative techniques, improved perioperative management, enhanced myocardial protection, and better postoperative care, have led to continued improvements in CABG outcomes [3]. Nonetheless, in-hospital mortality remains the most significant adverse outcome following isolated CABG. Although the reported in-hospital mortality rate is low [4], there is considerable variation among different medical centers [5]. Unlike mixed cardiac surgery cohorts, this population is relatively homogeneous concerning the surgical procedure. Additionally, its low baseline mortality creates a context where traditional prediction models may have limited effectiveness for individual patients. Therefore, a dynamic prediction model specifically designed for this population could identify fewer clinically relevant predictors while avoiding unnecessary complexity. Consequently, predicting rare outcomes, such as in-hospital mortality after elective isolated CABG, presents a considerable methodological challenge. Firth’s penalized likelihood approach can be utilized, as it effectively reduces estimation bias compared to standard logistic regression [6,7].

Traditionally, the European System for Cardiac Operative Risk Evaluation II (EuroSCORE II) is one of the most widely used tools for assessing risk prediction in cardiac surgery [8]. EuroSCORE II offers a static and incomplete preoperative assessment in CABG setting, as it does not consider the CAD severity, preoperative myocardial injury, anemia, any intraoperative or early postoperative complications. In a large cohort of CABG patients, EuroSCORE II showed satisfactory discriminative accuracy but significantly overestimated mortality risk, particularly for younger patients, as noted by Silverborn et al. [9]. This highlights the need to adjust the scoring system for different populations. Hijazi et al. proposed that integrating machine learning models with EuroSCORE II could improve predictions of post-CABG mortality [10]. Wu et al. demonstrated enhanced predictive performance by combining the triglyceride-glucose index with EuroSCORE II [11]. Ng et al. emphasized the need for EuroSCORE II to evolve by refining existing variables and adding new factors related to patient and procedural complexity [12]. These findings highlight the necessity of evaluating established risk models within specific contemporary surgical populations, rather than assuming that their predictive performance remains consistent across different institutions and patient groups.

Systemic inflammation has emerged lately as a significant factor influencing adverse outcomes following cardiac surgery. Processes such as cardiopulmonary bypass, surgical trauma, ischemia-reperfusion, and tissue injury initiate a complex inflammatory and immune response. This response can lead to endothelial dysfunction, myocardial injury, coagulation abnormalities, and postoperative organ dysfunction, particularly in patients with CAD, who already carry an additional inflammatory burden due to atherosclerosis. Consequently, composite inflammatory indices derived from peripheral blood cell counts are gaining recognition as cost-effective and readily accessible biomarkers for evaluating perioperative risk. More recent work further supports the potential clinical value of inflammatory indices in elective isolated on-pump CABG, such as NLR or SII, although their incremental value over established clinical risk models remains insufficiently characterized [13,14,15,16].

The present study was designed to address two complementary aspects of mortality prediction in patients undergoing elective isolated on-pump CABG. First, we explored to identify independent predictors of in-hospital mortality and to develop a parsimonious multivariable model specifically tailored to this low-event setting. We placed particular emphasis on integrating information available at various perioperative time points, thereby creating a dynamic prediction model rather than focusing solely on preoperative risk assessment. We hypothesized that a streamlined model, which combines baseline surgical risk with perioperative biological and hemodynamic information, could improve risk stratification in this context, where in-hospital mortality is an uncommon yet clinically significant outcome. Second, we examined the prognostic value of preoperative and early postoperative inflammatory indices for in-hospital mortality in this clinical context and evaluated whether these accessible biomarkers provide predictive insights beyond established clinical risk assessments. The identification of patients at risk for in-hospital death after elective isolated CABG is crucial for enhancing perioperative risk stratification, ensuring informed patient counseling, and optimizing postoperative monitoring, ultimately leading to better patient outcomes and efficient resource allocation.

## 2. Materials and Methods

### 2.1. Study Design

Our study had two objectives. First, we proposed to identify the independent risk factors and predictors of in-hospital mortality in patients undergoing elective isolated on-pump CABG surgery. We aimed to elaborate a parsimonious predictive model in this setting, tailored to a rare outcome, such as in-hospital mortality in elective isolated CABG. The endpoint was in-hospital mortality, defined as death occurring during the same hospitalization after surgery. Although we used a Firth-penalized logistic regression approach to reduce small-sample bias and the risk of separation, we acknowledge that it does not eliminate the limitations associated with the very low number of outcomes. Therefore, the resulting model should be considered exploratory and hypothesis-generating rather than a clinically validated prediction tool.

Secondly, we studied the predictive capacity of the preoperative and early postoperative inflammatory indices in this setting, compared to EuroSCORE II.

To achieve these objectives, we conducted a retrospective, single-center analysis, by reviewing the preoperative, intraoperative, and postoperative medical records of consecutive patients who underwent elective on-pump CABG at our tertiary center, the ‘Prof. C.C. Iliescu’ Emergency Institute for Cardiovascular Diseases in Bucharest, Romania, between 2022 and 2024. We included patients who underwent isolated on-pump CABG surgery, without any additional cardiac procedures, such as valvular surgery. The sample size was not predetermined; however, the consecutive selection approach minimized potential selection bias. We excluded patients who died during surgery, as well as those with incomplete data from our analysis. For handling missing data, we applied listwise deletion and analyzed the potential bias introduced by this method.

We developed three models tailored to different phases of the perioperative process: the Preoperative Model, Intraoperative Model, and Postoperative Model. The variables were carefully selected by evaluating their performance in univariable analysis, considering their clinical significance, and ensuring they adhered to standards for multicollinearity. This approach ensures that the chosen variables not only show strong individual associations but also contribute meaningful insights without excessive overlap in what they represent. To mitigate the risk of overfitting, we limited the selection to a maximum of three variables for evaluation in the multivariable logistic regression using Firth’s penalized likelihood method. The selected predictors were incorporated into two final multivariable models, which were explored for their ability to predict in-hospital mortality.

This study was conducted following the Declaration of Helsinki and was approved by the Ethics and Studies Approval Committee of the Prof. C.C. Iliescu Emergency Institute for Cardiovascular Diseases, Bucharest (No. 16112/1 July 2025). Patient consent was waived due to the retrospective design of the study.

### 2.2. Procedure

All patients followed the same perioperative protocol. They had previously been diagnosed with coronary artery disease and met the criteria for surgery. The Heart Team evaluated the patients using a multidisciplinary approach, which included a cardiac surgeon, a cardiologist, and a cardiac anesthesiologist along with intensive care specialists. In addition to coronary angiography, the preoperative evaluation included a comprehensive range of assessments, such as blood tests, transthoracic echocardiography, arterial Doppler studies, and screenings for both viral and bacterial infections, along with other personalized assessment. Any additional medical conditions were assessed using a multidisciplinary approach, in accordance with our institutional protocols. Subsequent to the preoperative assessment, the patients underwent on-pump CABG surgery. In some patients, carotid endarterectomy (CEA) was performed before CABG during the same procedure. This approach was implemented to mitigate the risk of perioperative stroke associated with coronary surgery, as determined by the Heart Team.

Full sternotomy in conjunction with normothermic CPB were performed in all surgeries. General anesthesia was used in all patients, along with standard and invasive monitoring techniques. An arterial catheter, a central venous catheter, and a urinary catheter were placed. Intraoperative transesophageal echocardiography was performed in all patients. Following the surgical procedure, patients were subsequently monitored and cared for in the intensive care unit (ICU).

### 2.3. Data Collection

We collected information on the following factors: sex, age, laboratory data, body mass index (BMI), and other comorbidities, including insulin-dependent diabetes mellitus, chronic obstructive pulmonary disease, the presence of the significant arteriopathy lesions and carotid atherosclerotic stenosis. EuroSCORE II was utilized as risk score to assess mortality risk [8]. During the intraoperative phase, we recorded the number of grafts performed, noted if CEA surgery was conducted simultaneously, and the use of the intra-aortic balloon pump (IABP). Additionally, we measured the duration of cardiopulmonary bypass and the time the ascending aorta was clamped. Postoperatively, we collected data related to the early vasoactive-inotropic score (VIS) [17] recorded upon admission to the ICU, incidents of death, and laboratory data. We used the following VIS formula: VIS = dobutamine dose (mcg/kg/min) + 100 × epinephrine dose (mcg/kg/min) + 10 × milrinone dose (mcg/kg/min) + 10,000 × vasopressin dose (U/kg/min) + 100 × norepinephrine dose (mcg/kg/min).

We reviewed preoperative and early postoperative laboratory data, at ICU admission. The collected data consisted of hematologic data, namely, hemoglobin concentration, leukocytes count, neutrophils count, lymphocytes count, monocytes count, platelets count, red cell distribution width—standard deviation (RDW-SD), platelet distribution width (PDW), the mean platelet volume (MPV), but also inflammatory indices, Creatine Kinase (CK), Creatine Kinase MB (CKMB), creatinine. We retrospectively calculated CKMB/CK ratio preoperatively and early postoperatively. The tested inflammatory indexes, aggregate index of systemic inflammation (AISI), systemic inflammatory index (SII), systemic inflammatory response index (SIRI), neutrophils to lymphocyte ratio (NLR), monocytes to lymphocyte ratio (MLR), and platelet to lymphocyte ratio (PLR), were retrospectively calculated. We used the following formulas: AISI = N × P × M/Lf; SII = N × P/Lf; SIRI = N × M/Lf; NLR = N/Lf; MLR = M/Lf; PLR = P/Lf. The preoperative creatinine clearance was calculated using the Cockcroft-Gault equation.

### 2.4. Statistical Analysis

All statistical analyses were performed using IBM SPSS Statistics version 30 (IBM Corp., Armonk, NY, USA) and R statistical software version 4.6.1. (R Foundation for Statistical Computing, Vienna, Austria). Descriptive statistics, between-group comparisons, univariable logistic regression analyses, assessment of multicollinearity, and ROC analyses were performed using SPSS.

We set a statistical significance threshold of 95% (*p* ≤ 0.05) and divided the cohort into two subgroups: Survivors and Non-Survivors. Categorical variables were presented as counts and percentages (n%) and analyzed using Fisher’s exact test. Quantitative variables were expressed as the median and interquartile range (IQR) and evaluated using the Mann–Whitney test to compare these variables between the two subgroups. To achieve our first objective, we began with univariable logistic regression analyses. We evaluated potential multicollinearity among the variables using variance inflation factors (VIFs) and considered clinical relevance when selecting variables for the multivariable analysis. Given the anticipated low number of in-hospital mortality events, we utilized multivariable logistic regression with Firth’s penalized likelihood, employing the logistf package in R. This approach helps to mitigate small-sample bias and addresses issues related to sparse data and separation.

To avoid overfitting, we selected a maximum of 3 variables to be assessed in multivariable logistic regression with Firth’s penalized likelihood, creating models corresponding to one perioperative moment: Preoperative Model, Intraoperative Model, Postoperative Model. The resulting predictors were included further in two final multivariable models (Model A and Model B). Subsequently, we assessed their relative performance (Model A and Model B). We assessed the variables that remain independent predictors in the final models and the models’ performance in Firth’s penalized likelihood [likelihood ratio test, AUC, clinical relevance].

The two models were compared in predicting in-hospital mortality. This assessment utilized a likelihood ratio test, comparisons of AUCs between models, and DeLong’s test. For the final model that showed the best performance, we evaluated its discrimination capacity, reporting the AUC, CI 95%, and providing a graphical representation of the ROC curve. Furthermore, we examined the model’s calibration by comparing predicted versus observed risks, illustrated in a calibration plot. We reported the calibration intercept, slope, and the Brier score. We performed internal validation in the same cohort using 1000 bootstrap resamples, reporting the optimism-corrected AUC. For the final model, individual predicted probabilities of in-hospital mortality were calculated from the estimated regression coefficients of the Firth-penalized logistic regression model. The predicted probability was obtained from the linear predictor using the logistic transformation, *p*$=1/\left[1+exp\left(-LP\right)\right]$, where LP represents the linear predictor derived from the model coefficients and individual predictor values. These predicted probabilities were subsequently used for risk stratification by dividing the cohort into five groups according to quintiles of the predicted mortality probability. A dataset-derived threshold corresponding to the upper-risk stratum was explored descriptively to identify patients with higher predicted mortality risk. This threshold was not prespecified or clinically validated. The clinical utility of the exploratory final model has not been evaluated in this study.

For our second objective, we conducted ROC analysis to assess the predictive capacity of preoperative and early postoperative inflammatory indices concerning the endpoint. This analysis allowed us to compare these variables with one another and with the risk score (EuroSCORE II) in the context of our study. We reported AUC, *p*-value, CI 95%, and cut-off values, along with their respective sensitivity (Ss) and specificity (Sp) when the AUC reached statistical significance. Additionally, we performed DeLong Test to test the AUCs difference significance.

## 3. Results

**Figure 1:**
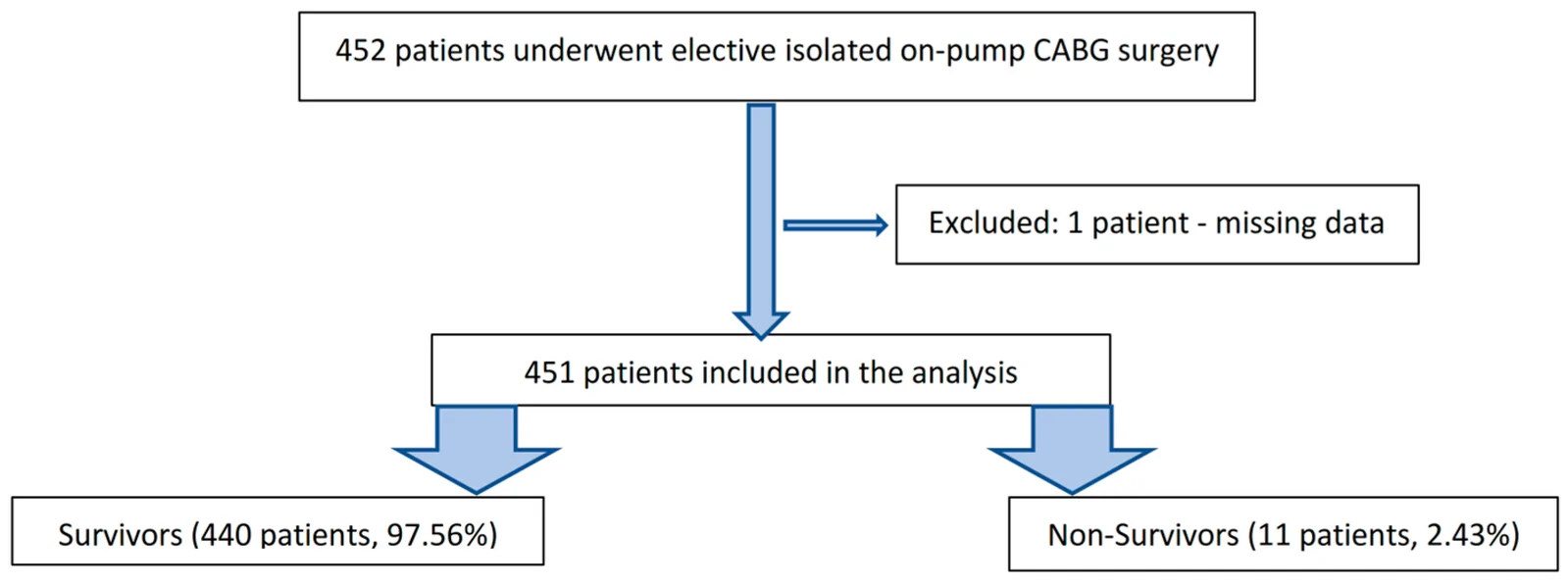
Flowchart of patient selection and in-hospital mortality outcomes.

We conducted a retrospective review of the medical data for 452 consecutive patients who underwent elective isolated on-pump CABG surgery between 2022 and 2024 at the Emergency Institute for Cardiovascular Diseases “Prof. C.C. Iliescu” in Bucharest, Romania. No patient died intraoperatively. Additionally, we chose to exclude one patient due to incomplete data. This decision introduced minimal bias in our analysis, as this excluded patient represented only 0.22% of the total sample. The diagram of the study was presented in Figure 1.

### 3.1. Data Presentation

We examined a cohort of 451 consecutive patients, comprising 78 females (17.29%) and 373 males (82.70%). We found that 11 patients out of the analyzed 451 patients died (2.43%). The perioperative data reviewed in the two subgroups: Survivors and Non-Survivors were presented in Table 1.

Subject to the analysis of two disproportionate subgroups, Non-Survivors had a significantly higher preoperative EuroSCORE II compared to Survivors (3.26 [1.31–4.14] versus 1.59 [1.08–2.24]). In our cohort, we found that 6.5% of female patients and 1.6% male patients have died, with female sex significantly associated with our endpoint (*p* = 0.027). Non-survivors exhibited higher preoperative anemia, but we found no additional mortality risk associated with preoperative BMI, insulin-dependent diabetes mellitus, chronic obstructive pulmonary disease diagnoses. Furthermore, Non-Survivors exhibited poorer renal function, as reflected by decreased creatinine clearance and elevated preoperative creatinine levels. Notably, we found no differences between the two groups in terms of age, preoperative inflammatory indices, CK, CKMB, CKMB/CK ratio, or other preoperative hematologic parameters, except for preoperative hemoglobin levels. Non-survivors experienced longer durations of cardiopulmonary bypass and aortic cross-clamping compared to survivors (Table 1). The number of grafts did not show significant differences, and performing CEA did not increased the risk of in-hospital mortality. IABP was notably linked to our endpoint. Postoperatively, VIS and postoperative NLR were significantly higher in Non-Survivors (112.8 [35–149.2] vs. 6 [2.5–12], respectively, 11.12 [8.32–14.86] vs. 7.81 [5.3–10.98]). Moreover, postoperative hemoglobin levels and platelet counts were significantly lower in non-survivors. Conversely, postoperative CK and CK-MB levels, but not their ratios, were significantly higher in the non-Survivors group.

### 3.2. First Objective: The Dynamic Prediction Model of In-Hospital Mortality

We evaluated each variable in univariable logistic regression analysis. The results of this analysis were presented in Table S1. The results of the Firth penalized logistic regression corresponding to Preoperative, Intraoperative and Postoperative Models were presented in Table S2.

#### 3.2.1. Preoperative Model

Among the preoperative variables, we found that only EuroSCORE II, sex, preoperative hemoglobin, preoperative creatinine clearance, preoperative creatinine, and preoperative CKMB/CK ratio yielded significant results in the univariable analysis (Table S1). EuroSCORE II includes details on sex and preoperative renal function, but lacks data on preoperative hemoglobin and CKMB/CK ratio. Therefore, we have strategically constructed the Preoperative Model: EuroSCORE II + hemoglobin + CKMB/CK. The model was significant (likelihood ratio test *p* = 0.0014). In the Firth penalized logistic regression model including preoperative variables, EuroSCORE II and preoperative CKMB/CK ratio were independently associated with in-hospital mortality. Higher EuroSCORE II was strongly associated with increased mortality risk (OR 1.54, CI 95% 1.14–2.08, *p* = 0.007), while elevated preoperative CKMB/CK was independently associated with higher in-hospital mortality (OR 17.57, CI 95% 1.19–223.07, *p* = 0.037). Preoperative hemoglobin was not independently associated with in-hospital mortality (OR 0.79, CI 95% 0.55–1.15, *p* = 0.204).

#### 3.2.2. Intraoperative Model

Among the intraoperative variables, only IABP, cardiopulmonary bypass duration and aortic cross-clamping time presented significant results in univariable analysis (Table S1). After testing for multicollinearity, we constructed the Intraoperative Model: IABP + cardiopulmonary bypass duration + aortic cross-clamping time. The model had significant likelihood ratio test (*p* = 0.001). In the Firth penalized logistic regression model including intraoperative variables, IABP use was independently associated with increased in-hospital mortality (OR 11.28, CI 95% 2.66–45.64, *p* = 0.002). Aortic cross-clamp time and cardiopulmonary bypass duration were not independently associated with mortality (*p* > 0.05).

#### 3.2.3. Postoperative Model

Among the postoperative variables recorded at ICU admission, only postoperative hemoglobin, postoperative platelet count, and VIS emerged with significant results in univariable logistic regression analysis (Table S1). After testing for multicollinearity, we constructed the Postoperative Model: VIS + hemoglobin + platelet count. In the Firth penalized logistic regression model including postoperative variables, VIS was independently associated with increased in-hospital mortality (OR 1.015, CI 95% 1.005–1.030, *p* = 0.001). The postoperative hemoglobin and platelet count were not independently associated with mortality (*p* = 0.613 respectively *p* = 1).

#### 3.2.4. Final Models

Variables emerged from preoperative, intraoperative and postoperative models were included in two final models (Table 2 and Table S3).

Model A included three variables corresponding to distinct perioperative moments: EuroSCORE II, IABP, and VIS. The model demonstrated good discriminatory performance for in-hospital mortality in our clinical setting (Likelihood ratio test 32.18, *p* = 1 × 10^−6^). EuroSCORE II was identified as an independent predictor of our endpoint (OR 1.55, CI 95%: 1.127–2.083, *p* = 0.011). Furthermore, VIS emerged as a highly significant independent predictor (OR 1.018, CI 95%: 1.005–1.032, *p* = 0.0005). In contrast, IABP did not maintain statistical significance (*p* = 0.155).

Model B consisted of three variables referring to preoperative assessment (preoperative risk and myocardial ischemic marker) and postoperative result of surgery (VIS at ICU admission): EuroSCORE II, preoperative CKMB/CK ratio, and VIS. The model demonstrated good discriminatory performance for in-hospital mortality in our clinical setting (Likelihood ratio test 32.18, *p* = 4.8 × 10^−7^). EuroSCORE II remained independent predictor (OR 1.57, CI 95%: 1.13–2.14, *p* = 0.011). CKMB/CK was independently associated with our endpoint (OR 21.06, CI 95%: 1.06–355.95, *p* = 0.046), but CI 95% was extremely large, probably due to small number of events (11 deaths). The very wide confidence interval indicated substantial uncertainty in the magnitude of this association. Accordingly, this result should be regarded as exploratory and interpreted cautiously. On the other hand, VIS was a very robust independent predictor (OR 1.017, CI 95% 1.008–1.033, *p* = 0.001).

#### 3.2.5. Discrimination and Internal Validation

The final Firth penalized logistic regression Model B demonstrated showed good discrimination in the study cohort (AUC 0.909, CI 95%: 0.795–1.000). All three variables remained independently associated with in-hospital mortality. Model A including IABP instead of preoperative CK-MB/CK ratio showed comparable discrimination (AUC 0.912, CI 95% 0.777–1.000) using DeLong test (*p* = 0.872, ∆AUC 0.0035, CI 95%: 0.039–0.046). After internal validation using 1000 bootstrap resamples, the optimism-corrected AUCs were 0.893 (Model A) and 0.892 (Model B), indicating similarly high internally validated discrimination.

#### 3.2.6. Calibration

Both models showed good apparent calibration. The calibration intercept and slope were −0.040 and 1.027 (Model A), and respectively, 0.130 and 1.080 (Model B). The corresponding Brier scores were 0.0198 and, respectively, 0.0204.

#### 3.2.7. Clinical Analysis

Only Model B retained all the three independent predictors, whereas IABP lost statistical significance after adjustment for baseline risk and postoperative hemodynamic support. Model B was retained as the final model for exploratory risk prediction, while Model A was used for sensitivity analysis. IABP was rather a marker of perioperative severity, its inclusion being more susceptible to confounding by indication. In this setting, the sensitivity Model A incorporating IABP was used to assess the robustness of the association between VIS and mortality.

#### 3.2.8. Model B (The Chosen Final Model) Assessment

**Figure 2:**
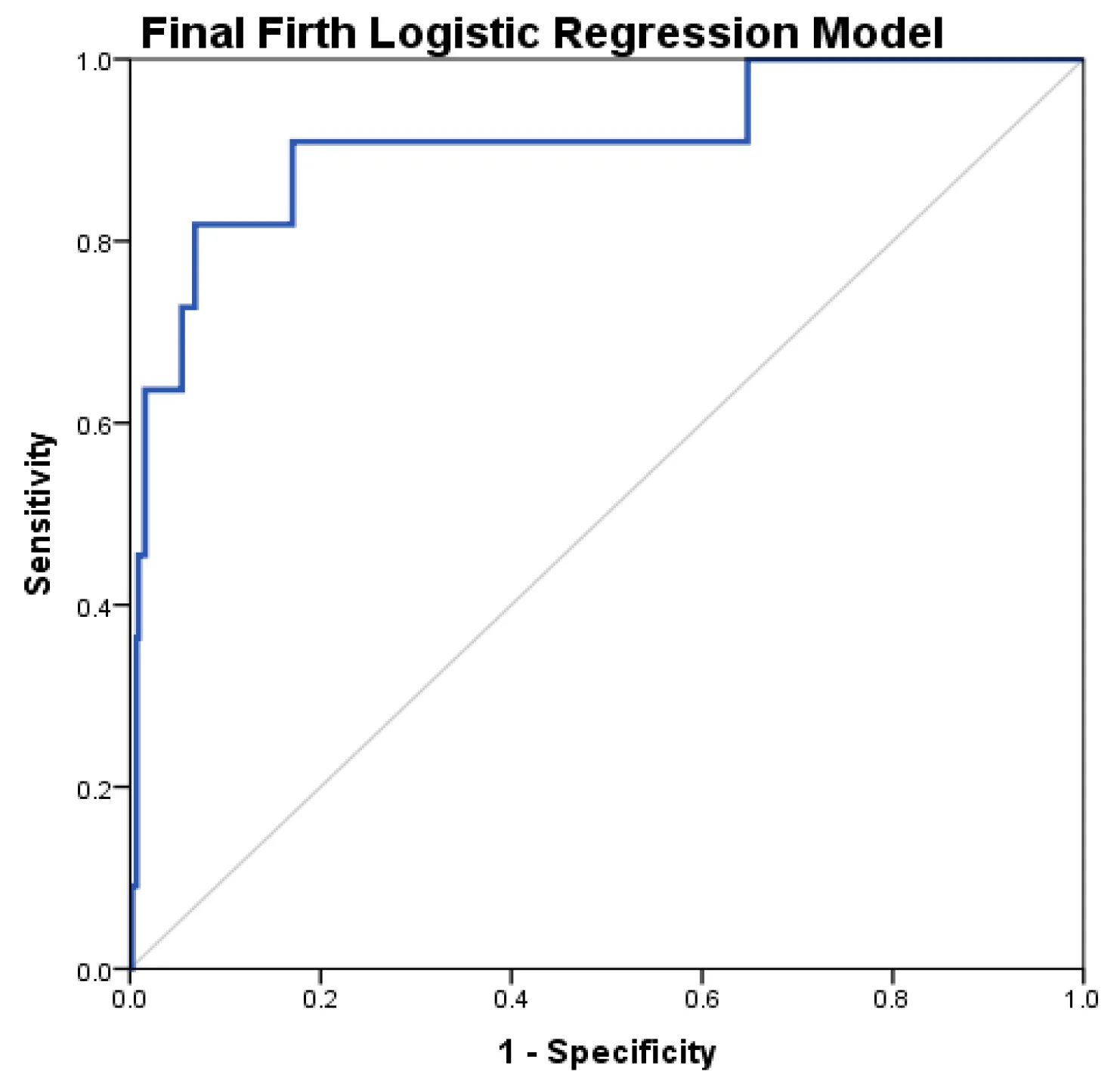
Receiver operating characteristic curve of the final Firth penalized logistic regression model including EuroSCORE II, preoperative CK-MB/CK ratio, and VIS at ICU admission. The area under the curve was 0.909 (CI 95% 0.795–1.000).

Model performance was assessed by discrimination (Figure 2) and calibration (agreement between predicted and observed mortality across quintiles of predicted risk).

**Figure 3:**
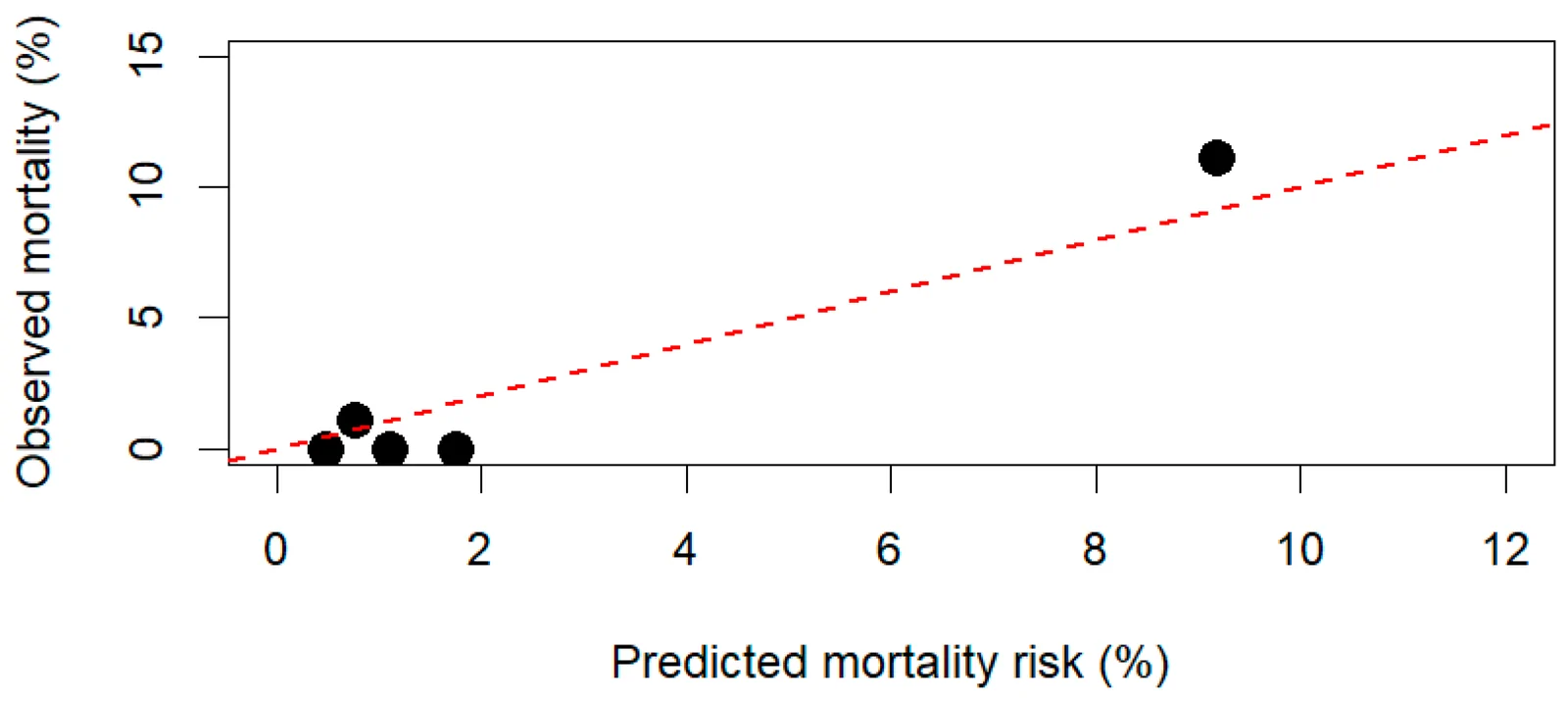
Calibration plot of the final Firth penalized logistic regression model (Model B). Predicted and observed mortality showed good agreement, with most observed events occurring in the highest predicted-risk group. Dots represent quintiles of predicted mortality risk. The dashed line indicates perfect calibration.

Calibration analysis demonstrated good agreement between predicted and observed mortality, with Brier Score 0.0204. The highest-risk group showed a predicted mortality of 9.2% compared with an observed mortality of 11.1%, containing 10 of the 11 observed deaths (Table 3, Figure 3).

The final Firth-penalized Model B included EuroSCORE II, VIS, and the preoperative CK-MB/CK ratio. Based on the estimated regression coefficients, the prediction equation for in-hospital mortality was:

$$logit\left(p\right)=-6.467+0.452\times EuroSCORE\;II+0.0163\times VIS+3.152\times CKMB/CK.$$

We calculated the predicted probability of in-hospital mortality:

$$p=\frac{1}{1+exp\left[-logit\left(p\right)\right]}.$$

The resulting predicted probabilities were used to stratify patients into five risk quintiles. The highest-risk quintile (Q5) comprised patients with a predicted mortality probability of ≥2.35%. This threshold was derived and evaluated within the same cohort and should not be interpreted as a clinically validated cutoff.

### 3.3. Second Objective: The Inflammatory Indices Analysis

We analyzed the ability of preoperative and early postoperative inflammatory indices to predict in-hospital mortality in elective on-pump isolated CABG. The results of the ROC analysis for these variables and EuroSCORE II are presented in the Table 4.

We found that none of the preoperative or postoperative inflammatory indices predicted our endpoint, except for the postoperative NLR. The postoperative NLR did demonstrate statistically significant, although modest, discriminatory ability for in-hospital mortality (AUC 0.684, 95% CI 0.527–0.840, *p* = 0.037). Importantly, postoperative NLR was also not significantly associated with in-hospital mortality in univariable regression analysis. Although EuroSCORE II showed a numerically higher AUC (AUC 0.750, CI 95%: 0.527–0.840, *p* = 0.037) than postoperative NLR, the difference between the two correlated ROC curves was not statistically significant (DeLong test, ∆ AUC 0.066, CI 95%: 0.183–0.315; *p* = 0.603).

## 4. Discussion

In this study involving 451 patients undergoing elective, isolated, on-pump CABG, we conducted a stepwise evaluation of various factors based on their timing during the perioperative period. Instead of limiting our analysis to established preoperative risk factors, we investigated whether intraoperative and early postoperative variables could provide additional prognostic insights. The final model included variables representing different stages of the patient’s surgical journey: EuroSCORE II, which is a recognized measure of preoperative risk; the CK-MB/CK ratio, serving as a preoperative biochemical marker; and the Vasopressor Inotrope Score (VIS) upon admission to the ICU, indicating early requirements for cardiovascular support after surgery. Given the low number of outcome events, the proposed model should be regarded as exploratory and hypothesis-generating rather than as a clinically validated prediction tool. Although the model demonstrated encouraging internal discrimination and calibration, these findings require confirmation in larger, independent cohorts before any clinical application can be considered.

Due to the relatively low number of mortality events in this cohort (11 out of 451), we employed Firth’s penalized logistic regression to minimize small-sample bias and enhance the stability of our estimates. The concentration of 10 of the 11 observed deaths within the highest predicted-risk quintile is noteworthy and suggests a potential role for the model in identifying a particularly high-risk subgroup. Nevertheless, given the limited number of events, this finding should be regarded as exploratory and requires external validation before a clinically actionable threshold can be established. Furthermore, our analysis revealed that none of the preoperative or postoperative inflammatory indices predicted the primary endpoint, with the exception of the postoperative NLR. However, postoperative NLR did not provide additional value beyond the Euroscore II in predicting the endpoint.

In our cohort, EuroSCORE II was significantly associated with mortality (OR 1.57, 95% CI 1.13–2.14; *p* = 0.011). This finding aligns with previous validation studies demonstrating the effectiveness of EuroSCORE II in patients undergoing isolated CABG [18,19]. EuroSCORE II incorporates demographic characteristics, comorbidities, cardiac function, and the urgency and complexity of the surgery, capturing a broad spectrum of baseline risk [8]. However, it is important to note that the score is static and incomplete [9,10,11,12]. The continued significance of EuroSCORE II in our final model indicates that baseline clinical risk remains highly relevant, even within a relatively homogeneous population restricted to elective isolated CABG. Additionally, the fact that EuroSCORE II remained a significant predictor alongside the CK-MB/CK ratio and postoperative VIS suggests that preoperative risk assessment alone does not fully account for the factors contributing to perioperative mortality.

While postoperative CK-MB elevation and CK-MB ratio have been extensively linked to mortality after CABG [20,21,22], there is significantly less information regarding the prognostic importance of the preoperative CK-MB/CK ratio. In our cohort, the preoperative ratio was independently associated with in-hospital mortality (OR 21.06, 95% CI 1.06–355.0; *p* = 0.046), suggesting that myocardial injury or vulnerability may already be reflected in circulating enzyme patterns before surgical intervention. However, caution should be exercised in interpreting the biological significance of the CK-MB/CK ratio. CK-MB is not completely specific to myocardial injury [23]. Additionally, in CAD patients, preoperative elevation of these enzymes may indicate a range of clinical and subclinical myocardial injuries rather than a single underlying pathological cause. Therefore, the CK-MB/CK ratio should be regarded as a potentially useful marker of perioperative risk rather than as a direct surrogate of myocardial viability or ischemic burden. The wide CI in our analysis indicated considerable uncertainty about the strength of the association, especially with only 11 observed deaths. As a result, the preoperative CK-MB/CK ratio should be viewed as an exploratory predictor rather than a solid prognostic factor. Its potential for improving perioperative risk prediction requires validation in larger studies that include modern cardiac biomarkers like high-sensitivity cardiac troponin. Unfortunately, high-sensitivity troponin measurements, which are more widely used and generally more specific to cardiac conditions than CK-MB, were not routinely available at our institution during the study period. As a result, we could not incorporate this variable into our analysis retrospectively. We emphasize the need for future prospective and multicenter studies to assess the incremental prognostic value of high-sensitivity troponin.

Nonetheless, we underscore the significance of evaluating non-cardiologic values for these biomarkers, including the CK-MB/CK ratio in patients with oncological conditions [24,25,26].

VIS at ICU admission demonstrated the strongest statistical association with mortality in the final model (OR 1.017, 95% CI 1.008–1.033; *p* < 0.001). VIS integrates the intensity of vasoactive and inotropic support required to maintain cardiovascular stability and may therefore provide an early, composite measure of postoperative cardiovascular dysfunction. The prognostic relevance of VIS is consistent with previous studies and meta-analyses demonstrating associations between higher postoperative VIS and adverse outcomes after cardiac surgery [27,28]. Importantly, the fact that VIS remained significant alongside EuroSCORE II suggests that early postoperative hemodynamic status may provide prognostic information that is not captured by preoperative risk assessment alone.

However, we did not collect data on preoperative medication, lactate levels, echocardiographic or electrocardiographic data, or intraoperative and postoperative arterial hypotension, all of which can predict postoperative vasoplegia and perioperative complications [29,30,31]. Preventing hypotension and hypoperfusion is essential to avoid organ dysfunction and mortality; however, the inotropic-vasopressors use can also elevate myocardial oxygen consumption, trigger cardiac arrhythmias, and lead to immune-mediated injuries. Moreover, they may cause peripheral, intestinal, and cardiac ischemia, which can be life-threatening [32,33]. Additionally, the use of catecholamines was linked to hyperglycemia, insulin resistance, immunosuppression, bacterial growth, increased virulence, and the formation of biofilms [32,33]. Therefore, a thoughtful combination of vasopressors, inotropes, and volume repletion is required, guided by advanced hemodynamic monitoring. An additional relevant finding was the ability of the final model to identify a small subgroup with markedly higher observed mortality. A predicted mortality probability of ≥2.35%, corresponding to the highest risk quintile, identified 90 patients and captured 10 of the 11 observed deaths. Conversely, patients below this threshold had an observed mortality of only 0.28%, corresponding to a negative predictive value (NPV) of 99.7%. Given that the 2.35% threshold was derived from the same dataset utilized for model development and evaluation, it should be regarded as a dataset-derived risk-stratification threshold rather than a clinically validated cutoff. Its effectiveness in concentrating the majority of observed deaths within the highest-risk group may, in part, reflect the specific characteristics of this cohort and the limited number of events. Likewise, the high observed NPV should be interpreted cautiously in view of the low overall mortality rate and the small number of observed events, with only one death occurring below the threshold. Thus, the NPV should be considered a descriptive, cohort-specific finding rather than evidence of clinically validated rule-out performance. External validation is necessary before this risk stratification can be considered for clinical application.

Numerous studies have highlighted the role of inflammatory indices in predicting outcomes during hospital stays for both cardiac [16,34,35,36] and non-cardiac surgeries [37,38,39]. In contrast, our analysis did not identify a consistent prognostic contribution from preoperative or postoperative inflammatory indices, with the exception of postoperative NLR. However, its discriminatory performance was modest and it was not significantly associated with mortality in univariable regression analysis. The association between postoperative NLR and mortality is biologically plausible, as the NLR reflects the balance between innate inflammatory activation and adaptive immune response and may increase substantially following major cardiac surgery. Nevertheless, the absence of significant improvement in discrimination when comparing the postoperative NLR to EuroSCORE II is an important finding.

An additional finding of our analysis was that IABP lost statistical significance after adjustment for baseline risk and postoperative hemodynamic support. Nevertheless, this interpretation should remain cautious because IABP use is subject to confounding by indication. Patients receiving IABP are inherently different from those who do not require mechanical circulatory support, and the decision to initiate IABP depends on clinical circumstances that may not be completely captured by the variables included in the model. Several randomized trials and meta-analyses in high-risk CABG have suggested a mortality benefit from prophylactic preoperative IABP, including analyses restricted to isolated on-pump CABG. Conversely, observational studies have reported increased mortality among patients receiving IABP, highlighting the potential influence of confounding by indication [40,41,42,43].

The main strength of this study is the focus on a relatively homogeneous CABG population and the integration of preoperative and early postoperative variables into the same prognostic framework. The use of Firth penalization is another important methodological strength, given the low number of mortality events and the potential for conventional logistic regression to produce biased or unstable estimates under these conditions.

Taken together, our findings support a dynamic model of perioperative risk assessment. EuroSCORE II captures baseline patient and procedural risk, preoperative CK-MB/CK ratio may provide additional information regarding the preoperative myocardial biochemical profile, and admission VIS reflects the early postoperative cardiovascular response. Conversely, although postoperative NLR showed an association with mortality, it did not significantly improve discrimination beyond EuroSCORE II. This suggests that not all statistically associated biomarkers necessarily provide clinically meaningful incremental prognostic information. Such a sequential approach could help identify patients who may benefit from closer hemodynamic monitoring, earlier optimization of cardiovascular support, and more intensive postoperative surveillance.

We developed an integrated prognostic model specifically for this cohort, rather than focusing on identifying previously unknown individual risk factors. Our proposed model has the potential to distinguish between patients with a particularly low risk of in-hospital mortality and those with a higher predicted risk. The 2.35% threshold we established serves as a model-derived risk-stratification point and should not be interpreted as a validated clinical cutoff. The current study did not prospectively evaluate clinical utility, nor did it establish predefined clinical decision thresholds or net clinical benefit analyses. As a result, the observed optimism-corrected AUC of 0.892 and the favorable calibration results should be interpreted as evidence of strong internal model performance rather than as proof of clinical applicability. To facilitate clinical implementation, external validation and prospective evaluations of clinically relevant decision thresholds and net clinical benefit are essential. However, the present findings should be considered hypothesis-generating until confirmed in larger and independent cohorts.

Several limitations should be acknowledged. This study is a single-center retrospective analysis of a relatively small cohort. Although 451 consecutive patients undergoing isolated on-pump CABG were included over a predefined 2-year period, only 11 postoperative deaths occurred. We did not predetermine the sample size; instead, we used a consecutive patient approach to minimize potential selection bias. While missing data could introduce additional biases, its overall impact on the study was negligible. It is important to report the disproportionality between the groups studied (survivors and non-survivors). All patients in our analyzed cohort underwent on-pump CABG; however, some also had additional carotid surgery. Although model complexity was deliberately restricted and Firth penalization and bootstrap internal validation were used, these approaches do not eliminate the limitations imposed by the small number of outcome events. This limited event rate may affect the stability and generalizability of the multivariable model and increases the potential risk of overfitting. Accordingly, the present model should be considered exploratory and hypothesis-generating, and external validation in larger, multicenter cohorts with a higher number of outcome events is warranted before clinical implementation. For this reason, rather than proposing a definitive clinical threshold based on the present cohort, we have emphasized that the observed high-risk stratification should be considered hypothesis-generating and requires validation in an independent external cohort. Accordingly, the observed associations should not be interpreted as definitive evidence of independent causal relationships. Additionally, the prognostic value of the preoperative CK-MB/CK ratio and ICU admission VIS requires external validation, ideally in larger multicenter cohorts with sufficient numbers of mortality events. Furthermore, high-sensitivity cardiac troponin was not routinely available during the study period and therefore could not be evaluated. Given its widespread clinical use and greater cardiac specificity compared with CK-MB, future studies should assess whether incorporating high-sensitivity troponin provides incremental prognostic value. We did not include information on preoperative symptoms. Moreover, there were institutional limitations that impacted our analysis, including inconsistencies in data regarding various inflammatory indicators, such as levels of protein C and fibrinogen. We also lacked data on the types of grafts and their patency, as well as whether the revascularization was complete. We also lacked detailed information on intraoperative transfusion requirements, nadir intraoperative hemoglobin concentrations, and data on hemodynamic and volumetric status. Additionally, we did not have information on preoperative aggregability tests or treatments. Our analysis relied solely on RDW-SD, as RDW-CV data was inconsistently available. Other important factors, such as troponin, BNP, albumin, lactate levels, electrocardiography, and echocardiographic data, were also missing from our analysis. These limitations hindered our ability to conduct a more comprehensive evaluation. Furthermore, our study lacks external validation of the findings, which should be carefully taken into account when interpreting the results. To further validate our findings, it is essential to conduct future prospective multicenter studies.

## 5. Conclusions

A perioperative model combining EuroSCORE II, preoperative CK-MB/CK ratio, and early postoperative VIS demonstrated encouraging internal discrimination and calibration for in-hospital mortality after elective isolated on-pump CABG. The potential prognostic contribution of the CK-MB/CK ratio requires confirmation in larger, independent cohorts. The highest-risk quintile, defined using a dataset-derived predicted mortality threshold of ≥2.35%, contained 10 of the 11 observed deaths. Given the low number of mortality events, these findings should be considered exploratory and hypothesis-generating, and the proposed threshold should not be interpreted as a clinically validated cutoff. External validation is therefore essential before the model or its dataset-derived risk stratification can be considered for clinical application.

## Figures and Tables

**Table 1:** Clinical and perioperative characteristics of survivors and non-survivors.

Variable	Survivors (*n* = 440)	Non-Survivors (*n* = 11)	*p* ^1^
PREOPERATIVE			
Age (years) ^2^	66 [59–70]	66 [63–73]	0.274
EuroSCORE II ^2^	1.59 [1.08–2.24]	3.26 [1.31–4.14]	0.005
Male sex ^3^	367 (83.40%)	6 (54.54%)	0.027
BMI (kg/m^2^) ^2^	27.22 [23.8–30.84]	27.20 [25–33.2]	0.559
Insulin-dependent diabetes mellitus ^3^	45 (10.22%)	2 (18.18%)	0.321
Chronic obstructive pulmonary disease ^3^	143 (32.5%)	5 (45.45%)	0.352
Significant peripheral arteriopathy ^3^	108 (24.54%)	4 (36.36%)	0.478
Significant carotid stenosis ^3^	140 (31.81%)	5 (45.45%)	0.343
Creatinine clearance (mL/min) ^2^	79.41 [64.55–97.87]	50.11 [40.62–86.81]	0.020
Leukocyte count (×10^3^) ^2^	7.87 [6.65–9.11]	7.40 [6.63–10.23]	0.996
Hemoglobin (g/dL) ^2^	13.9 [13–14.87]	12.5 [11.7–13.7]	0.033
Platelet count (×10^3^) ^2^	248.5 [201.25–291]	237 [219–272]	0.911
Neutrophil count (×10^3^) ^2^	4.73 [3.84–5.73]	4.41 [3.75–6.66]	0.935
Lymphocyte count (×10^3^) ^2^	2.13 [1.63–2.65]	2.29 [1.59–2.67]	0.856
Monocyte count (×10^3^) ^2^	0.62 [0.51–0.79]	0.7 [0.41–0.88]	0.592
RDW-SD (fl) ^2^	41.75 [39.8–44.4]	41.5 [40.3–43.5]	0.846
PDW (%) ^2^	12.4 [11.2–13.8]	12.9 [12.1–15.9]	0.108
MPV (fl) ^2^	10.5 [9.92–11.10]	10.7 [10.2–12]	0.183
Creatinine (mg/dL) ^2^	0.96 [0.83–1.13]	1.2 [0.93–1.41]	0.023
CK (UI/L) ^2^	80.5 [57–116]	48 [34–99]	0.101
CKMB (UI/L) ^2^	24 [19–33]	30 [16–36]	0.519
CKMB/CK ratio ^2^	0.32 [0.22–0.43]	0.37 [0.29–0.79]	0.109
AISI ^2^	345.92 [226.44–527.42]	356.48 [238.09–597.47]	0.811
SII ^2^	546.73 [342.65–804.49]	476.42 [375.88–963.46]	0.907
SIRI ^2^	1.38 [0.97–2.01]	1.57 [1–2.01]	0.937
NLR ^2^	2.18 [1.63–3.14]	2.24 [1.66–2.57]	0.872
PLR ^2^	117.84 [92.16–149.16]	118.78 [82.02–189.29]	0.773
MLR ^2^	0.29 [0.23–0.38]	0.31 [0.27–0.37]	0.830
INTRAOPERATIVE			
Number of grafts ^2^	3 [2–3]	3 [3–3]	0.615
Concomitant carotid surgery ^3^	19 (4.31%)	2 (18.18%)	0.088
IABP ^3^	28 (6.36%)	4 (36.36%)	0.005
Cardiopulmonary bypass duration (min) ^2^	91 [74–110]	126 [91–190]	0.002
Aortic cross-clamp time (min) ^2^	52 [42–68]	70 [62–98]	0.005
EARLY POSTOPERATIVE			
VIS ^2^	6 [2.5–12]	112.8 [35–149.2]	0.001
Leukocyte count (×10^3^) ^2^	13.16 [9.9–17.24]	12.13 [10.58–14.46]	0.596
Hemoglobin (g/dL) ^2^	10.25 [9.3–11.2]	8.6 [8.4–9.8]	0.001
Platelet count (×10^3^) ^2^	168 [137.25–212.75]	105 [91–179]	0.012
Neutrophil count (×10^3^) ^2^	10.67 [7.89–14.15]	10.56 [9.63–12.83]	0.992
Lymphocyte count (×10^3^) ^2^	1.37 [0.98–1.88]	0.94 [0.87–1.72]	0.054
Monocyte count (×10^3^) ^2^	0.73 [0.46–1.06]	0.58 [0.31–0.7]	0.123
RDW-SD (fl) ^2^	41.65 [39.9–44.4]	42.5 [42.1–43.7]	0.094
PDW (%) ^2^	12 [10.9–12.4]	12.4 [11–14.6]	0.481
MPV (fl) ^2^	10.5 [10–11.2]	11.3 [11.3–11.9]	0.157
Creatinine (mg/dL) ^2^	0.83 [0.71–0.99]	1.07 [0.9–1.23]	0.022
CK (UI/L) ^2^	322.5 [235–459]	380 [328–932]	0.020
CKMB (UI/L) ^2^	60.5 [46–89]	93 [76–141]	0.008
SII ^2^	1330.74 [843.51–2028.19]	1147.94 [809.13–2579.79]	0.996
SIRI ^2^	5.92 [2.73–10.76]	5.57 [4.48–7.7]	0.818
AISI ^2^	991.92 [456.29–1920.26]	731 [483.74–1393.09]	0.257
PLR ^2^	124.56 [92.49–167.22]	88.35 [77.1–265.96]	0.638
MLR ^2^	0.53 [0.32–0.85]	0.56 [0.35–0.64]	0.844
NLR ^2^	7.81 [5.3–10.98]	11.12 [8.32–14.86]	0.037
CKMB/CK ratio ^2^	0.19 [0.16–0.24]	0.21 [0.13–0.25]	0.692

^1^, the result of the exact Fisher test or Mann-Whitney test; ^2^, data presented as median (IQR); ^3^, data presented as n (%). Abbreviations: AISI, aggregate index of systemic inflammation.; BMI, body mass index; CK, creatine phosphokinase; CKMB, myocardial form of creatine phosphokinase; IABP, intra-aortic balloon pump; MLR, monocytes-to lymphocytes ratio; MPV, mean platelet volume; NLR, neutrophils-to lymphocytes ratio; PDW, platelets distribution width; PLR, platelets-to lymphocytes ratio; RDW-SD, red cell distribution width-standard deviation; SII, systemic immune-inflammation index; SIRI, systemic inflammation response index; VIS, vasoactive-inotropic score.

**Table 2:** The results of the Firth penalized logistic regression analysis in the final models.

Model	Variable	*p*	Adjusted OR	CI 95%
Model A	EuroScore II	0.011	1.55	1.127–2.083
	IABP	0.155	3.38	0.586–14.270
	VIS	0.00057	1.018	1.005–1.032
Model B	EuroScore II	0.011	1.571	1.13–2.14
	VIS	0.00002	1.017	1.008–1.033
	Preoperative CKMB/CK ratio	0.046	21.06	1.06–355

Abbreviations: CI, confidence interval; IABP, intra-aortic balloon pump; OR, odds ratio; CKMB/CK, creatine phosphokinase-MB/creatine phosphokinase ratio; VIS, vasoactive-inotropic score.

**Table 3:** Calibration table (Model B).

Risk Group	n	Average Predicted Risk	Observed Mortality	Non-Survivors
1	91	0.48%	0%	0
2	90	0.76%	1.11%	1
3	90	1.10%	0%	0
4	90	1.75%	0%	0
5	90	9.19%	11.1%	10

**Table 4:** ROC analysis -inflammatory indices and EuroSCORE II (endpoint in-hospital mortality).

Variable	*p*	AUC (CI 95%)	Cut-Off	Sensitivity	Specificity
EuroSCORE II	0.005	0.750 (0.583–0.917)	2.77	63.6%	84.8%
PREOPERATIVE					
AISI	0.811	0.521	-	-	-
SII	0.907	0.510			
SIRI	0.937	0.507			
NLR	0.872	0.486			
PLR	0.773	0.525			
MLR	0.830	0.519			
EARLY POSTOPERATIVE					
AISI	0.257	0.400			
SII	0.996	0.500			
SIRI	0.818	0.480			
NLR	0.037	0.684 (0.527–0.840)	9.98	72.7%	67.3%
PLR	0.638	0.458			
MLR	0.844	0.483			

Abbreviations: AISI, aggregate index of systemic inflammation; MLR, monocytes-to lymphocytes ratio; NLR, neutrophils-to lymphocytes ratio; PLR, platelets-to lymphocytes ratio; SII, systemic immune-inflammation index; SIRI, systemic inflammation response index.

## Data Availability

The data presented in this study are available on request from the corresponding author, due to privacy and ethical restrictions.

## Supplementary Materials

The following supporting information can be downloaded at: https://www.mdpi.com/article/10.3390/jcm15187202/s1, Table S1: The results of the univariable binary logistic regression analysis (in-hospital death endpoint); Table S2: The results of the Firth penalized logistic regression analysis upon Preoperative, Intraoperative and early Postoperative multivariable models; Table S3: Characteristics of Model A and Model B.
