# Supplementary material for: Dynamic Perioperative Risk Stratification of In-Hospital Mortality After Elective Isolated On-Pump CABG: A Single-Center Firth Penalized Logistic Regression Analysis
Source: J Clin Med. 2026 Sep 16;15(18):7202. doi: 10.3390/jcm15187202 (PMC13607704; doi:10.3390/jcm15187202)
Supplement: Supplementary file 1 [file jcm-15-07202-s001.zip › jcm-4535803-supplementary.pdf]

Table S1. The results of the univariable binary logistic regression analysis (in-hospital death endpoint)

Variable	Univariable Analysis		
	P [1]	Exp(B)	OR (CI 95%)
**PREOPERATIVE**			
**Age** [2]	0.135		
**EuroSCORE II** [2]	0.002	1.621	1.621 (1.195-2.198)
**Male sex** [3]	0.021	4.189	4.189 (1.245-14.093)
**BMI** [2]	0.241		
Insulin-dependent diabetes mellitus [3]	0.352		
Chronic obstructive pulmonary disease [3]	0.321		
**Significant peripheral arteriopathy**[3]	0.478		
**Significant carotide stenosis** [3]	0.343		
Creatinine clearance (ml/min) [2]	0.020	0.966	1.035 (1.005-1.064)
Leukocyte count (*10^3) [2]	0.443		
Hemoglobin (g/dl)[2]	0.049	0.700	1.428 (1.002-2.036)
Platelet count (*10^3) [2]	0.619		
Neutrophil count (*10^3) [2]	0.480		
Lymphocyte count (*10^3) [2]	0.761		
Monocyte count (*10^3) [2]	0.249		
**RDW-SD** [2]	0.658		
**PDW** [2]	0.156		
**MPV** [2]	0.157		
Creatinine (mg/dl) [2]	0.025	4.271	4.271 (1.203-15.168)
**CK** (UI/l) [2]	0.531		
**CKMB** (UI/l) [2]	0.922		
**CKMB/CK ratio** [2]	0.016	22.259	22.259 (1.804-274.69)
**AISI** [2]	0.999		
**SII** [2]	0.333		
**SIRI** [2]	0.827		
**NLR** [2]	0.338		
**PLR** [2]	0.698		
**MLR**[2]	0.750		
**INTRAOPERATIVE**			
**Number of grafts** [2]	0.647		
**Concomitant carotide surgery** [3]	0.051		
**IABP** [3]	0.001	8.408	8.408 (2.322-30.445)
Cardiopulmonary bypass duration (min) [2]	0.001	1.023	1.023 (1.011-1.034)
Aortic cross-clamp time (min) [2]	0.001	1.031	1.031 (1.013-1.049)
**EARLY POSTOPERATIVE**			
**VIS** [2]	0.001	1.026	1.026 (.013-1.039)
Leukocyte count (*10^3) [2]	0.431		
Hemoglobin (g/dl) [2]	0.001	0.419	2.386 (1.412-4.032)
Platelet count (*10^3) [2]	0.028	0.985	1.015 (1.002-1.028)
Neutrophil count (*10^3) [2]	0.667		
Lymphocyte count (*10^3) [2]	0.087		
Monocyte count (*10^3) [2]	0.174		
RDW-SD (fl) [2]	0.152		
PDW (%)[2]	0.615		
MPV (fl)[2]	0.214		
Creatinine (mg/dl)[2]	0.060		
**CK** (UI/l) [2]	0.061		
**CKMB** (UI/l) [2]	0.128		

**SII** [2]	0.998
**SIRI** [2]	0.429
**AISI** [2]	0.233
**PLR** [2]	0.784
**MLR** [2]	0.458
**NLR** [2]	0.140
**CKMB/CK ratio**[2]	0.549

[1], the result of the univariable analysis; [2], data presented as median (IQR); [3], data presented as n (%). Abbreviations: AISI, aggregate index of systemic inflammation; BMI, body mass index; CK, creatine phosphokinase; CKMB, myocardial form of creatine phosphokinase; IABP, intra-aortic balloon pump; MLR, monocytes-to lymphocytes ratio; MPV, mean platelet volume; NLR, neutrophils-to lymphocytes ratio; PDW, platelets distribution width; PLR, platelets-to lymphocytes ratio; RDW-SD, red cell distribution width-standard deviation; SII, systemic immune-inflammation index; SIRI, systemic inflammation response index; VIS, vasoactive-inotropic score;

Table S2. The results of the Firth penalized logistic regression analysis upon Preoperative, Intraoperative and early Postoperative multivariable models

Variable	Likelihood ratio test	Firth penalized logistic reggression		
	p	*p*	**Adjusted OR**	**CI 95%**
**PREOPERATIVE MODEL**				
**EuroSCORE II**	0.0014	0.007	1.54	1.195-2.198
**Hemoglobin**		0.204	1.26	0.86-1.81
**CKMB/CK ratio**		0.037	17.57	1.19-223.07
**INTRAOPERATIVE MODEL**				
**IABP**	0.001	0.001	11.279	2.663-45.64
Cardiopulmonary bypass duration		0.687	1.006	0.972-1.032
Aortic cross-clamp time		0.196	1.025	0.988-1.076
**POSTOPERATIVE MODEL**				
**VIS**	0.001	0.001	1.017	1.006-1.032
**Hemoglobin**		0.079	0.603	0.328-1.058
**Platelet count**		0.408	0.994	0.979-1.007

Abbreviations: CI, confidence interval; IABP, intra-aortic balloon pump; OR, odds ratio; CKMB/CK, creatine phosphokinase-MB/creatine phosphokinase ratio; VIS, vasoactive-inotropic score

Table S3. Characteristics of Model A and Model B

Model	Likelihood ratio test	AUC (CI 95%)	Corrected AUC	Calibration intercept	Calibration Slope	Brier
**Model A**	32.18	0.912 (0.777-1)	0.893	-0.040	1.027	0.0198
**Model B**	32.18	0.909 (0.795-1)	0.892	0.130	1.080	0.0204
